# Effects of multiple cations and sintering temperature on microstructure and dielectric properties in Na_1/2_Ln_1/2_Cu_3_Ti_4_O_12_ (Ln = Sm and Eu) ceramic materials

**DOI:** 10.1038/s41598-023-42610-3

**Published:** 2023-09-15

**Authors:** Long-Fei Yuan, Ting Zhang, Dan-Dan Han

**Affiliations:** 1 College of Physics and Electronic Science, Zunyi Normal University, Zunyi, 563002 Guizhou China; 2 College of Chemistry and Chemical Engineering, Zunyi Normal University, Zunyi, 563002 Guizhou China; 3 College of Chemistry and Pharmaceutical Engineering, Jilin Institute of Chemical Technology, Jilin, 132022 Jilin China

**Keywords:** Ceramics, Solid-state chemistry

## Abstract

Na_1/2_Eu_1/2_Cu_3_Ti_4_O_12_ and Na_1/2_Sm_1/2_Cu_3_Ti_4_O_12_ dielectric ceramics were synthesized at different sintering temperatures (950, 975 and 1000 °C) by a solid-state reaction method. Phase structure, cation valence state, and dielectric properties of all sintered ceramics were systematically investigated. When the preparation temperature was changed, the Cu^+^ ion concentration of (Na^+^, Eu^3+^) co-doped ceramics changed faster than that of (Na^+^, Sm^3+^) co-doped ceramics. Abnormally high dielectric constants of ~ 3.17 × 10^4^ and ~ 1.06 × 10^4^ (at 10 Hz and 303 K) were achieved in Na_1/2_Sm_1/2_Cu_3_Ti_4_O_12_ and Na_1/2_Eu_1/2_Cu_3_Ti_4_O_12_ ceramics prepared at 950 °C, respectively. However, Na_1/2_Sm_1/2_Cu_3_Ti_4_O_12_ and Na_1/2_Eu_1/2_Cu_3_Ti_4_O_12_ prepared in high sintering temperature (1000 °C) exhibited a good frequency stability of dielectric permittivity. It was demonstrated that an increasing number of charge carriers induced by the increase of sintering temperature could lead to a competitive coexistence of two polarization mechanisms (surface barrier layer capacitor and internal barrier layer capacitor), further changing the dielectric properties of CaCu_3_Ti_4_O_12_-based ceramics.

## Introduction

With the rapid development of network and information technology, the fifth generation (5G) network, online classroom and other technologies that are inseparable from people’s lives are increasingly demanding the performance of capacitors, the basic components of electronic products^1–3^. Therefore, the research on the dielectric properties of different dielectric materials has always been a hot issue^4–7^. As a traditional dielectric material, CaCu_3_Ti_4_O_12_ (CCTO) has been closely watched since its giant dielectric properties were found^8–11^. Two major aspects ways are used to optimize the dielectric properties of CCTO-like ceramics: (1) use of different preparation methods, for instance, solid-state reaction method^12^, sol–gel^13^, and spark plasma sintering^14^, (2) the substitution of ions in the lattice, like La_2_O_3_^15^, Y_2/3_Cu_3_Ti_4_O_12_^16^,Mg_0.05_Al_0.05_^17^, Na_1/2_Y_1/2_Cu_3_Ti_4_O_12_^18^, Na_1/2_La_1/2_Cu_3_Ti_4_O_12_^19^, Ca_1-*x*_Sr_*x*_Cu_3_Ti_4_O_12-*x*_F_*x*_^20^ and Na_1/3_Yb_1/3_Ca_1/3_Cu_3_Ti_4_O_12_^21^, etc. Simultaneously, internal barrier layer capacitors (IBLC), as a major polarization mechanism, is used to explain the origin of dielectric behaviors for CCTO-based family materials^22,23^.

The IBLC model is considered to be an internal capacitor composed of semiconductor grains surrounded by insulating grain boundaries, which has also been discovered in other dielectric materials, such as the BaTiO_3_^−^ type ceramics^24^ and rutile TiO_2_-related ceramics^25,26^. Among them, the conductivity level of grain and grain boundary plays an important role in IBLC effect, which is affected by the number of charge carriers in the ceramic. For example, a high dielectric constant and a low loss tangent observed in Na_1/3_Ca_1/3_Bi_1/3_Cu_3_Ti_4_O_12_ has been attributed to the electrically heterogeneous and comprised of semiconducting grains and insulating grain boundaries, while the semiconducting grains is primarily due to electron hopping between Cu^+^ ↔ Cu^2+^ and Ti^3+^ ↔ Ti^4+^ sites^27^. The colossal dielectric of Na_1/2_Y_1/2_Cu_3_Ti_4_O_12_ ceramic are closely related to the electrically heterogeneous microstructure at the insulating grain boundary, and the origin of semiconducting grains is likely correlated with the Cu_2_O phase and the large amount of Ti^3+^/Ti^4+^ ions^28^. For (Al^3+^,Nb^5+^) co-doped CaCu_3_Ti_4_O_12_ ceramics, the high dielectric permittivities with good temperature stability has been attributed to the internal barrier layer capacitor model of Schottky barriers at grain boundaries, meanwhile electron hopping can occur in both Ti^3+^–O–Ti^4+^ and Cu^+^–O–Cu^2+^ ions^29^.

It is obvious that the dielectric properties of CCTO-related ceramics can be effectively improved by the doping of ions, especially multiple cations (shown in the Table 1). But few reports explained the relationship between the preparation conditions (especially sintering temperature), grain conductivity, internal polarization mechanism, and dielectric properties of ceramics. Thus, it is fundamentally important to build a clear picture for the evolution of internal polarization mechanism along with the number of charge carriers in grain. Up to the present time, little work has been done on the sintering temperature, structural and dielectric properties of the signal phase (Na^+^, Eu^3+^) or (Na^+^, Sm^3+^) co-doped CCTO ceramics, specifically Na_1/2_Eu_1/2_Cu_3_Ti_4_O_12_ and Na_1/2_Sm_1/2_Cu_3_Ti_4_O_12_. Boonlakhorn et al. and Somphan et al. reported the good dielectric properties of Na_1/2_Sm_1/2_Cu_3_Ti_4_O_12_ ceramics respectively, but a little impurity phase of Sm_4_Ti_3_O_12_ or CuO was detected in ceramics^31,32^. Considering the comparison with Sm doped ACu_3_Ti_4_O_12_ ceramics, the Eu ion closest to Sm in the periodic table of the elements was selected as doping ion to study the polarization mechanism and dielectric properties for CCTO-related ceramics. And, there is no discussion on the relationship between sintering temperature, the number of charge carriers, polarization mechanism and dielectric properties of Na_1/2_Eu_1/2_Cu_3_Ti_4_O_12_ and Na_1/2_Sm_1/2_Cu_3_Ti_4_O_12_ ceramics. A better understanding of the effect of polarization mechanism could be helpful for designing oxide ceramics with superior dielectric properties.

**Table 1:** Sintering process, mean grain size and dielectric properties of some LnCu_3_Ti_4_O_12_ (Ln = Na_1/2_Bi_1/2_, Na_1/2_Sm_1/2_, Na_1/2_Eu_1/2_, Na_1/2_La_1/2_, Na_1/2_Y_1/2_, Na_1/3_Ca_1/3_Bi_1/3_ and Na_1/3_Ca_1/3_Yb_1/3_ etc.) ceramics.

Samples	Preparation method	Sintering conditions	Mean grain size (μm)	Dielectric constant (1 kHz)	Dielectric loss (1 kHz)	References
Bi_1/2_Na_1/2_Cu_3_Ti_4_O_12_	Pechini method	1000 °C for 10 h	3.2	2.77 × 10^3^	0.11	^30^
Na_1/2_Bi_1/2_Cu_3_Ti_4_O_12_	Sol–gel	1000 °C for 3 h	2.2	≈ 10^4^	–	^9^
Na_1/3_Ca_1/3_Bi_1/3_Cu_3_Ti_4_O_12_	Solid state method	1060 °C for 5 h	2–20	2.59 × 10^4^	0.038	^27^
Na_1/2_Sm_1/2_Cu_3_Ti_4_O_12_	Sol–gel	1050 °C for 18 h	3.67	8.04 × 10^3^	0.045	^31^
Na_1/2_Sm_1/2_Cu_3_Ti_4_O_12_	Solid state method	1110 °C for 10 h	–	7.02 × 10^3^	0.041	^32^
Na_1/2_La_1/2_Cu_3_Ti_4_O_12_	Solid state method	1080 °C for 5 h	2–4	6.1 × 10^3^	0.037	^33^
Na_1/2_La_1/2_Cu_3_Ti_4_O_12_	Spark plasma sintering	925 °C for 10 min	0.3	> 10^3^	–	^14^
Na_1/2_Y_1/2_Cu_3_Ti_4_O_12_	Solid state method	1085 °C for 5 h	11	1.4 × 10^4^	0.04	^18^
Na_1/2_Y_1/2_Cu_3_Ti_4_O_12_	Solid state method	1110 °C for 10 h	10.32	1.8 × 10^4^	0.03	^28^
Na_1/2_Y_1/2_Cu_3_Ti_4.1_O_12_	Solid state method	1090 °C for 5 h	4.06	2.56 × 10^4^	0.022	^8^
(Na_1/3_Ca_1/3_Yb_1/3_)Cu_3_Ti_4_O_12_	Sol–gel	1075 °C for 12 h	0.18–0.4	5488	0.075	^21^
Na_1/2_Sm_1/2_Cu_3_Ti_4_O_12_	Solid state method	1000 °C for 10 h	19.73	5.5 × 10^3^	0.055	This work
Na_1/2_Eu_1/2_Cu_3_Ti_4_O_12_	Solid state method	1000 °C for 10 h	16.9	4.7 × 10^3^	0.066	This work

In this study, the Na_1/2_Ln_1/2_Cu_3_Ti_4_O_12_ (Ln = Eu or Sm) ceramics were synthesized by solid-state reaction. The relationship among microstructure, polarization mechanism and dielectric properties of Na_1/2_Ln_1/2_Cu_3_Ti_4_O_12_ (Ln = Eu or Sm) ceramics had been conformed for the first time by carefully tuning the number of charge carriers in grain via the change of sintering temperature. It could be notable that the sintering temperature altered the number of charge carriers in grain, induce different polarization mechanism, resulting in varied dielectric properties of the Na_1/2_Ln_1/2_Cu_3_Ti_4_O_12_ ceramics.

## Materials and methods

### Sample preparation

Na_1/2_Eu_1/2_Cu_3_Ti_4_O_12_ and Na_1/2_Sm_1/2_Cu_3_Ti_4_O_12_ ceramics were prepared via a solid state reaction method. The raw materials, TiO_2_ (99.99%), CuO (99.95%), CaCO_3_ (99.9%), Eu_2_O_3_ (99.9%) and Sm_2_O_3_ (99.9%) were carefully weighed and milled. First, the green pellets obtained with a uniaxial press were calcined at 600 °C for 10 h in air. The calcined pellets were ground into powders, and the obtained powders were pressed into pellets again. Then, the ceramic samples were obtained by sintering compacted powder at different temperatures from 950 to 1000 °C for 10 h. For clarity, the obtained ceramics are named based on the composition (Na_1/2_*X*_1/2_Cu_3_Ti_4_O_12_ expressed as N*X*CTO), and the sintering temperature (T in °C) as N*X*CTO-T. For example, the Na_1/2_Eu_1/2_Cu_3_Ti_4_O_12_ ceramic sintered at 950 °C is named as NECTO-950.

### Characterization

Crystal structure of these samples characterized using Powder X-ray diffraction (XRD) with a D8-focu Bruker apparatus (Cu Kα, λ = 0.15418 nm). The structural study was further performed by Raman spectra (RS) using a T64000 instrument under a 532 nm laser beam. The microstructural texture, and oxidation states were also analyzed by canning electron microscope (SEM) (FEI XL-30, America) and X-ray photoelectron spectroscopy (XPS, Escalab 250XI), respectively. The C1*s* photoemission line (284.6 eV) was used to calibrate the positions of the photoelectron spectra.

All pellets were carefully polished, and then coated surfaces with an Au layer film using a LJ-16 sputter coating unit. The dielectric properties of the ceramics were measured using a dielectric spectrometer (Novocontrol Concept 41) over the frequency and temperature ranges of 10 to 2 ⨯ 10^7^ Hz and − 70 to 200 °C.

## Results and discussion

### X-ray diffraction analysis

**Figure 1:**
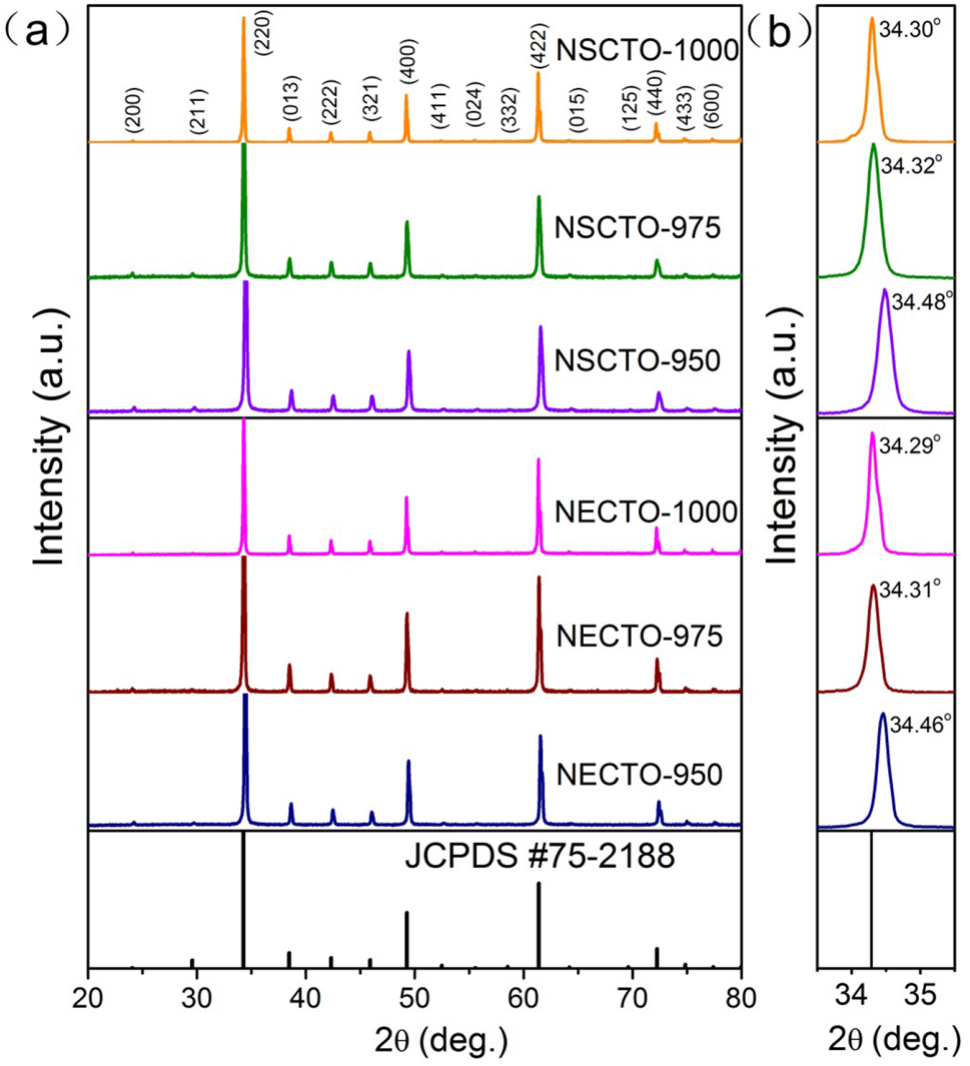
(**a**) XRD patterns of Na_1/2_Eu_1/2_Cu_3_Ti_4_O_12_ and Na_1/2_Sm_1/2_Cu_3_Ti_4_O_12_ ceramics sintered under different temperature; (**b**) changes of (220) diffraction peaks of these ceramic samples.

Sintered Na_1/2_Eu_1/2_Cu_3_Ti_4_O_12_ and Na_1/2_Sm_1/2_Cu_3_Ti_4_O_12_ ceramics were first characterized using an XRD technique to confirm its phase structures. Figure 1a demonstrates the XRD patterns of the NECTO and NSCTO ceramics sintered under various temperatures from 950 to 1000 °C. A main phase of CaCu_3_Ti_4_O_12_ (JCPDS 75-2188) with a cubic structure was defected in all ceramic samples. No impurity phase was observed. As shown in the Fig. 1b, the (220) peak shifts to a lower degree with increasing sintered temperature in the NECTO and NSCTO samples. The relevant lattice parameters, *a*, were calculated from the XRD patterns. The *a* values of the CaCu_3_Ti_4_O_12_ was 7.391 Å^21^. The *a* values of the NECTO and NSCTO ceramics sintered at 950 °C were respectively found to be 7.379 and 7.381 Å, respectively. The lattice parameters (*a*) of NECCTO-950 and NSCCTO-975 were slightly smaller than that of CaCu_3_Ti_4_O_12_, which was due to the small difference in the ionic radii in Na^+^ ($r_{{{\text{Na}}^{ + } }}$ = 1.39 Å), Sm^3+^ ($r_{{{\text{Sm}}^{3 + } }}$ = 1.24 Å), Eu^3+^ ($r_{{{\text{Eu}}^{3 + } }}$ = 1.12 Å) and Ca^2+^ ($r_{{{\text{Ca}}^{2 + } }}$ = 1.34 Å).

Although the radius of Na^+^ ion is larger than that of Ca^2+^, the lattice of NECCTO-950 and NSCCTO-950 sample shrined with the occupation of Sm^3+^ and Eu^3+^ ions with a smaller radius in the CCTO-type structure. The *a* values of the NECTO and NSCTO samples sintered at 975 and 1000 °C were 7.388, 7.399 and 7.387, 7.392 Å, respectively. Interestingly, *a* values of the NECTO and NSCTO samples sintered at high temperature were significantly larger than that of ceramics prepared at low temperature, which could be attributed to the change in the cation valence state in the rutile crystal structure.

### Raman spectrogram analysis

**Figure 2:**
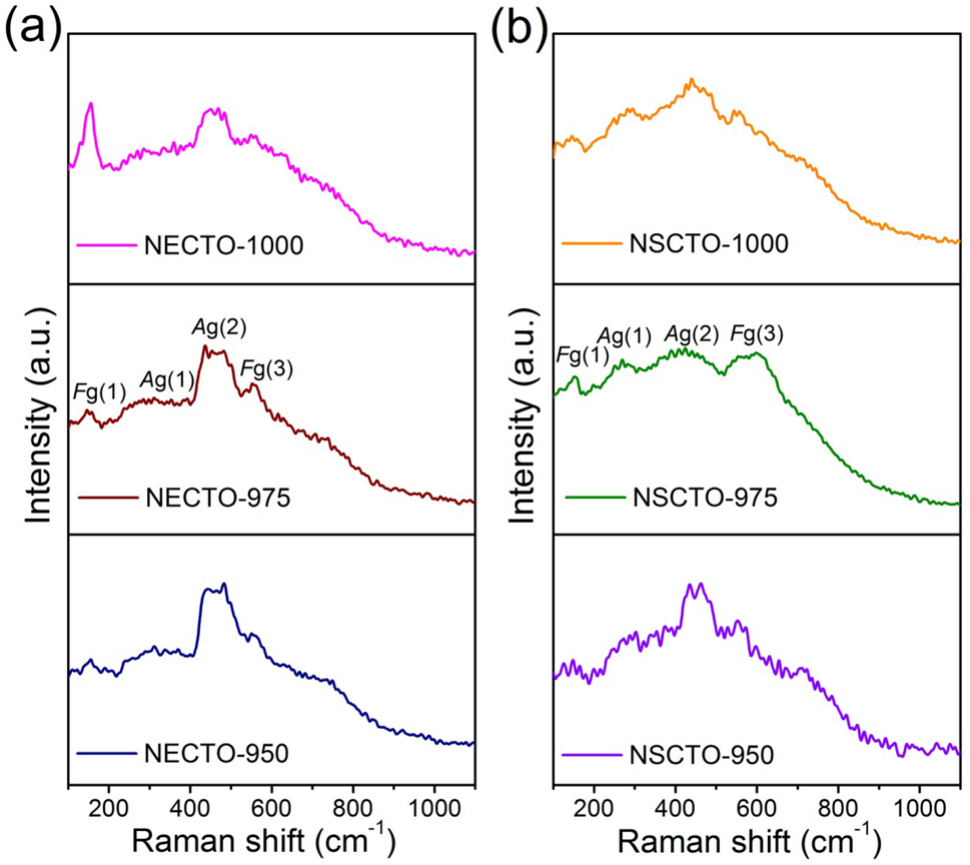
Raman spectra of (**a**) Na_1/2_Eu_1/2_Cu_3_Ti_4_O_12_ and (**b**) Na_1/2_Sm_1/2_Cu_3_Ti_4_O_12_ ceramics samples prepared in different sintering temperatures.

Raman spectra of NECTO and NSCTO pellets sintered at different temperature are depicted in Fig. 2a,b. In Fig. 2, five modes at around 153, 358, 450 and 552 cm^−1^ were observed for six ceramics, in agreement with previous reports^10^. The Raman active modes of NECTO and NSCTO ceramics, like CaCu_3_Ti_4_O_12_, could be similarly expressed as Γ_Raman_ = 2*A*_g_ + 2*E*_g_ + 4*F*_g_^34^. These Raman modes corresponded to *F*_g_ (1), *A*_g_ (1), *A*_g_ (2) and *F*_g_ (3), respectively. Among these modes, the *A*_g_ (1), *A*_g_ (2) and *F*_g_ (1) modes was attributed to TiO_6_ rotation like lattice vibrations, and the *F*_g_ (3) mode was associated with an O–Ti–O antistretching lattice vibrations.

### Microstructure

**Figure 3:**
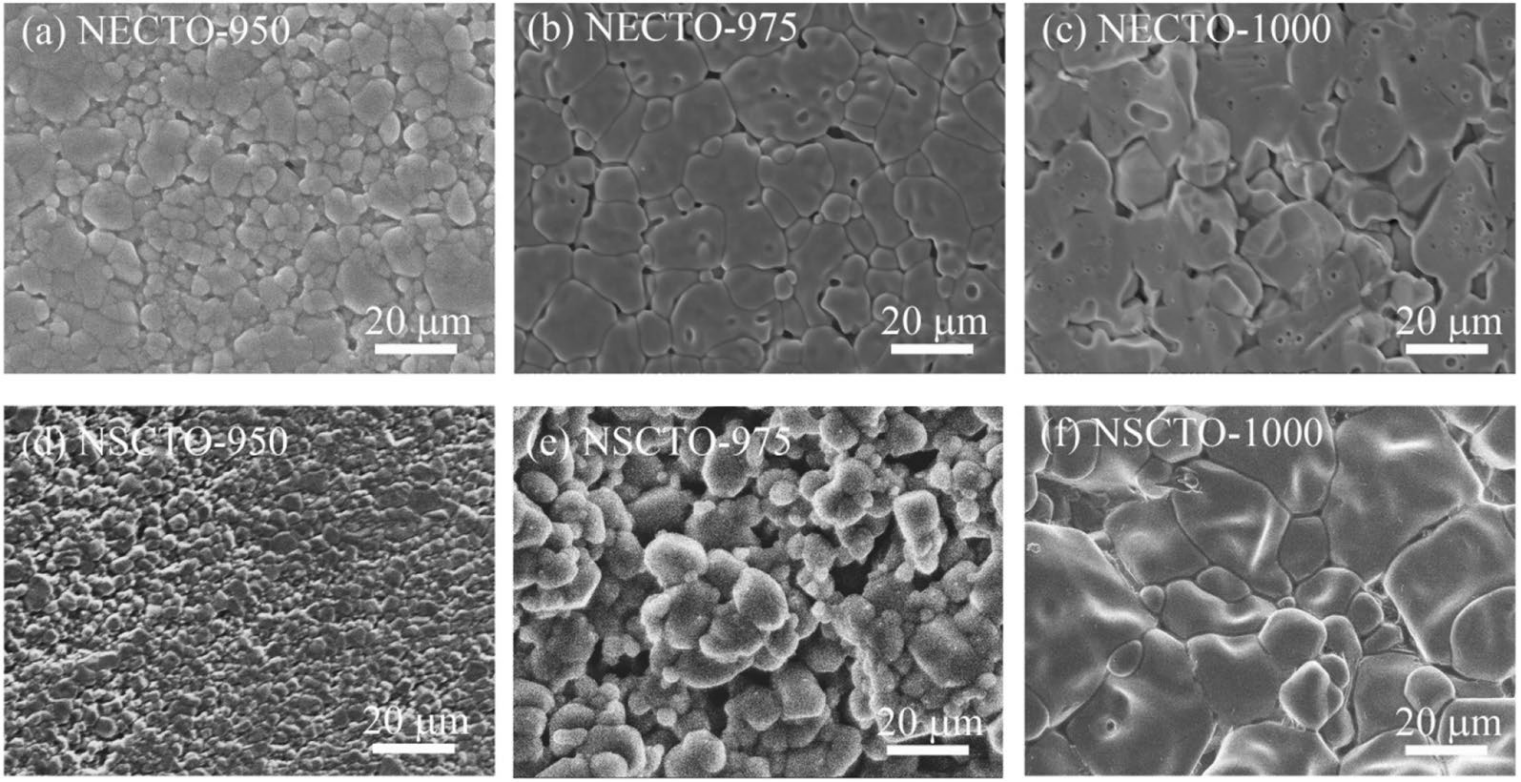
SEM images of Na_1/2_Eu_1/2_Cu_3_Ti_4_O_12_ and Na_1/2_Sm_1/2_Cu_3_Ti_4_O_12_ ceramics sintered under various temperature: (**a**) NECTO-950, (**b**) NECTO-975, (**c**) NECTO-1000, (**d**) NSCTO-950, (**e**) NSCTO-975 and (**f**) NSCTO-1000.

**Figure 4:**
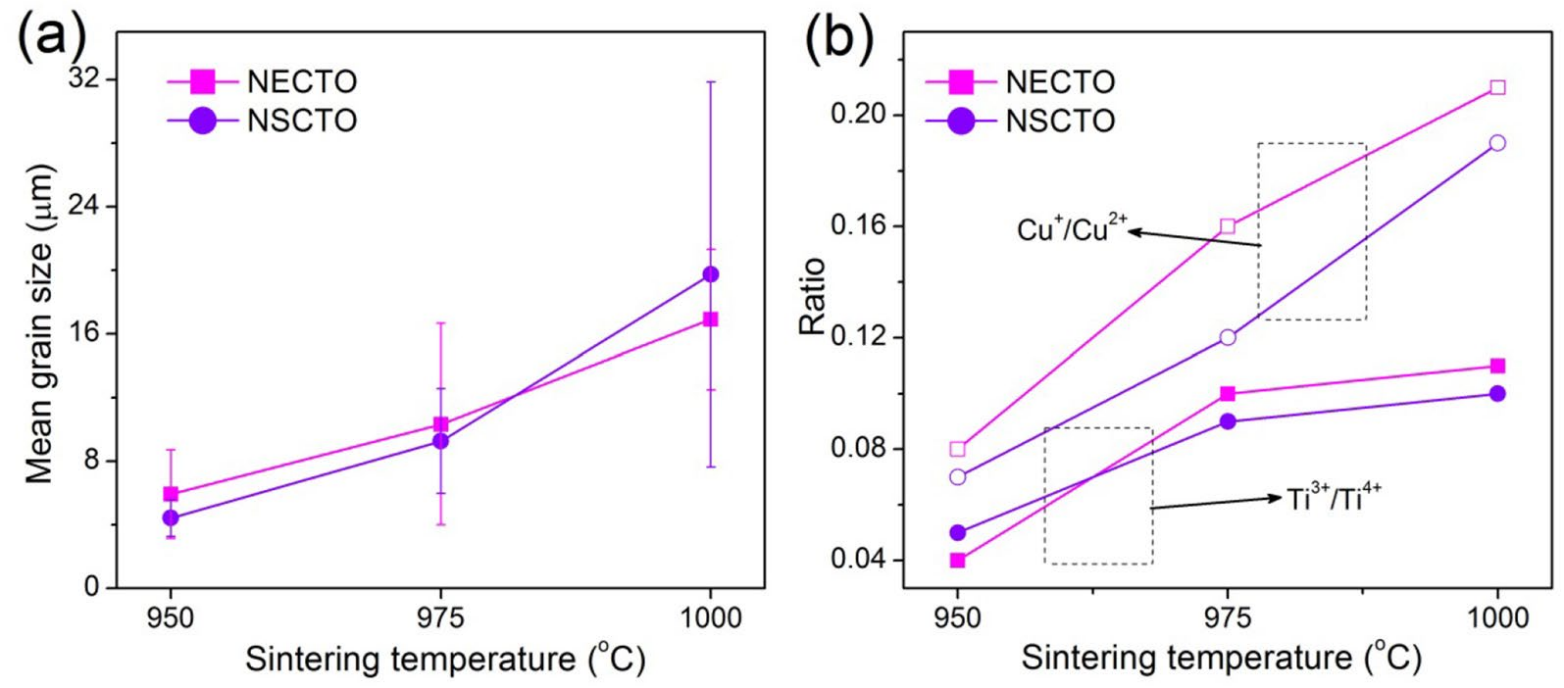
(**a**) Curves of mean grain size vs. sintering temperature for Na_1/2_Eu_1/2_Cu_3_Ti_4_O_12_ and Na_1/2_Sm_1/2_Cu_3_Ti_4_O_12_ ceramics. (**b**) Relationship between the atomic ratio of Cu^+^/Cu^2+^ or Ti^3+^/Ti^4+^ and sintering temperature for these samples. Solid dots and hollow dots in (**b**) correspond to the ratio of Ti^3+^/Ti^4+^ and the ratio of Cu^+^/Cu^2+^, respectively.

The microstructures of the NECTO-950, NECTO-975, NECTO-1000, NSCTO-950, NSCTO-975 and NSCTO-1000 are shown in Fig. 3a–f, respectively. The mean grain size of all the ceramics are revealed in Fig. 4a. Figure 3 shows that all the ceramic samples had a relatively dense microstructure. Grains and grain boundaries were clearly observed. However, a very small amount of pores appeared on the microscopic surface for the ceramic samples sintered in the low temperature (e.g. NECTO-950, NECTO-975, NSCTO-950 and NSCTO-975). To study the microscopic surface for the NECTO and NSCTO ceramics sintered at different temperature, as shown in Fig. 4a, it was found that the sintering temperature had a significant influence on the microstructure. The results showed that the mean grain size of Na_1/2_Eu_1/2_Cu_3_Ti_4_O_12_ samples prepared at various temperature increased slightly as the sintering temperature increased from 950 to 1000 °C, ≈ 5.93, ≈ 11.32, ≈ 16.9 μm, respectively. Similarly, the mean grain size of NSCTO ceramics also increased with the increase of sintering temperature. The change in the grain size of the ceramics is usually associated with the liquid phase sintering mechanism, as reported in other CCTO-related ceramics^30,35–37^. However, in case of the NSCTO ceramics, the microstructure largely changed when the sintering temperature changed by only 25 °C. This phenomenon was similar to the Na_1/2_Ln_1/2_Cu_3_Ti_4_O_12_-type ceramics reported by other researchers (shown in the Table 1). When the doping elements of Na_1/2_Ln_1/2_Cu_3_Ti_4_O_12_-type ceramics changed from Y, La, Sm, Yb to Bi, the grain size of the prepared ceramics also showed significant changes. It may be related to the state of the extranuclear electron of the element. The Sm element is one atomic number lower than the Eu element, and the electrons of the 4f electron orbit are also one less, which may result in different grain growth trends of the NSCTO and NECTO ceramics under the same preparation conditions. The density of the samples has been measured by Archimedes method. As sintering temperature increased, the relative density of the NECTO ceramics increased monotonically from 83% for T = 950 °C and 87% for T = 975 °C to 91% for T = 1000 °C. And the relative density of NSCTO ceramics also increased with sintering temperature, reaching 81%, 85% and 92%, respectively.

### XPS analysis

**Figure 5:**
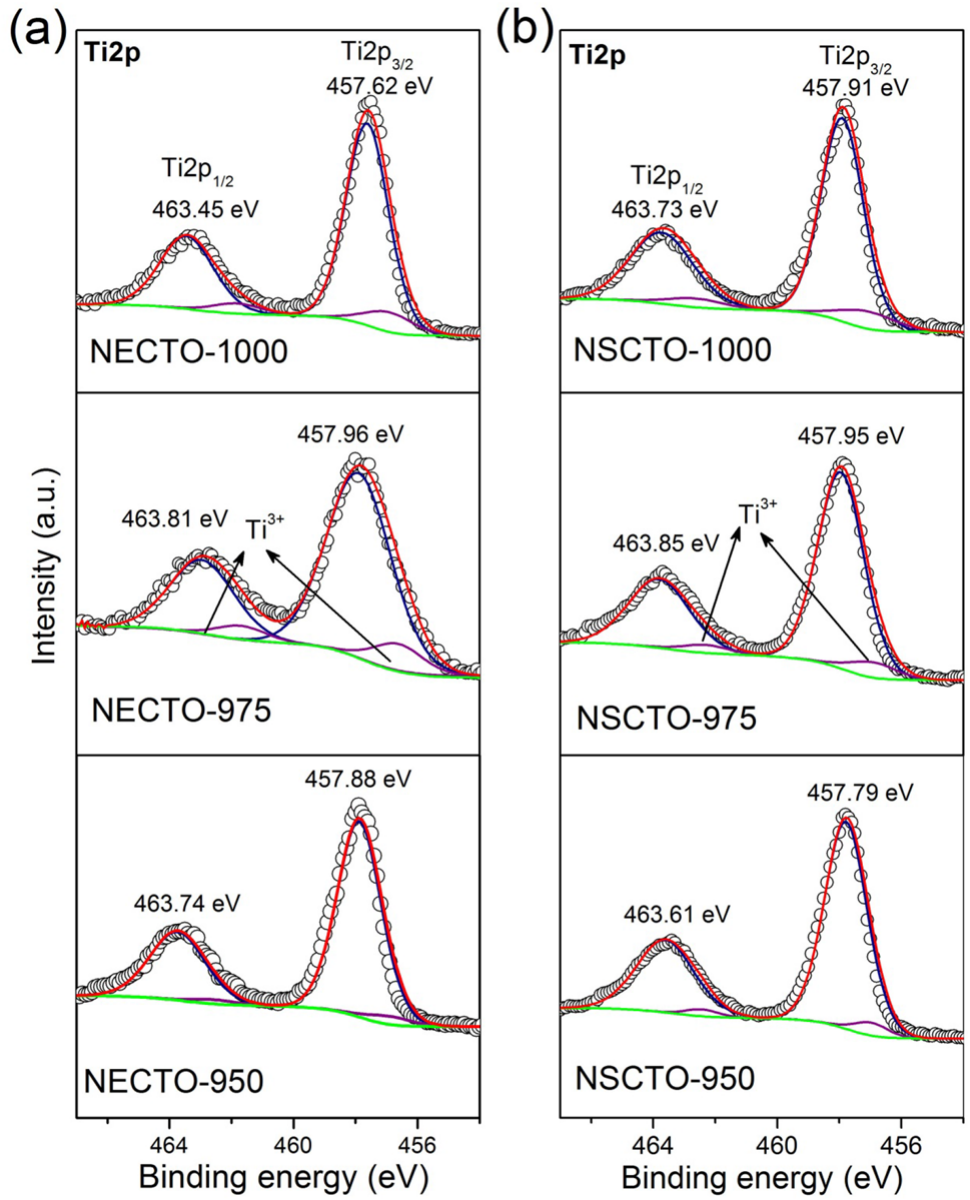
XPS spectra for the Ti 2*p* region for the (**a**) Na_1/2_Eu_1/2_Cu_3_Ti_4_O_12_ and (**b**) Na_1/2_Sm_1/2_Cu_3_Ti_4_O_12_ ceramics prepared at different sintering temperature.

**Figure 6:**
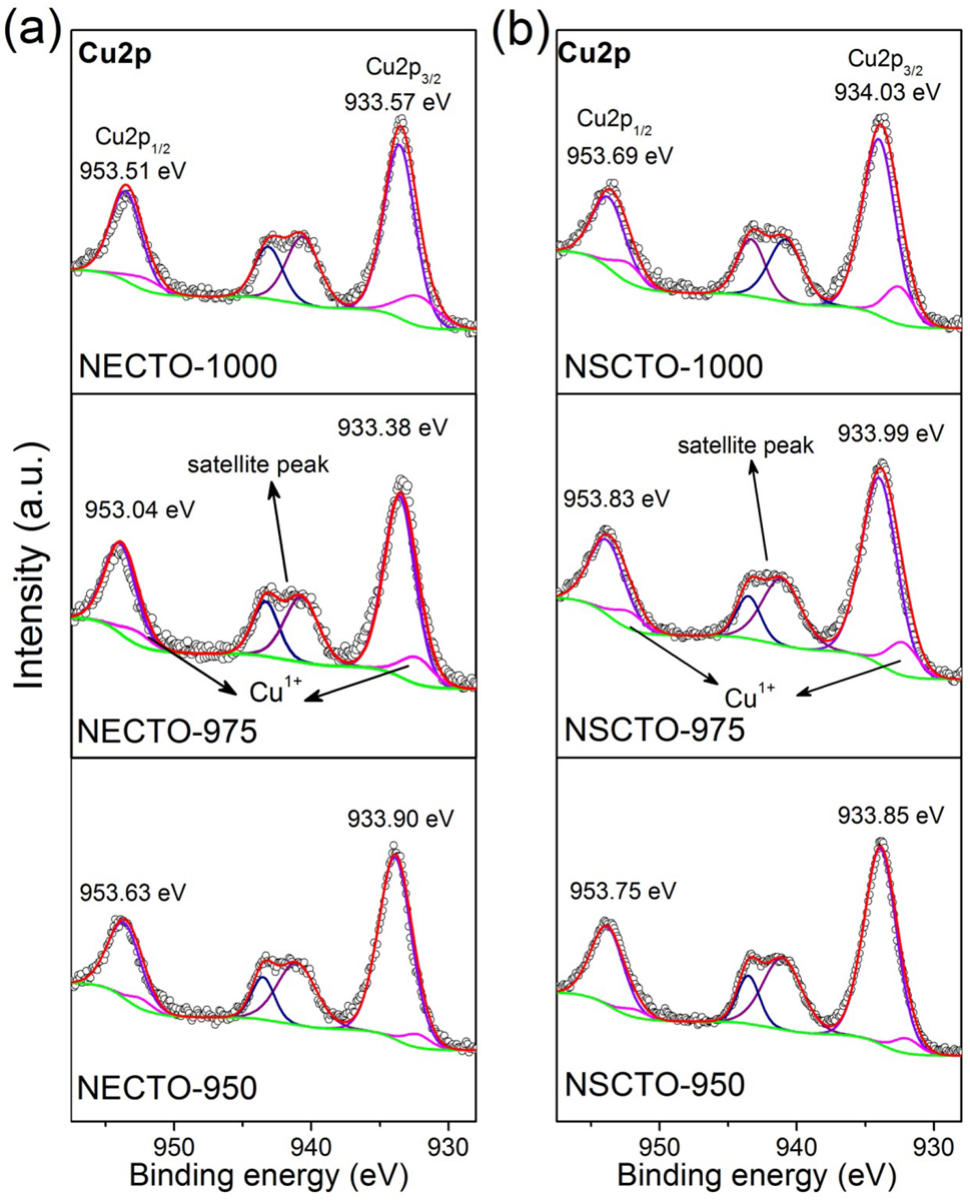
XPS spectra for the Cu 2*p* region for the (**a**) Na_1/2_Eu_1/2_Cu_3_Ti_4_O_12_ and (**b**) Na_1/2_Sm_1/2_Cu_3_Ti_4_O_12_ ceramics prepared at different sintering temperature.

To identify the oxidation states of the polyvalent cations, the XPS spectrum was corrected. Figures 5 and 6 displayed the spectra of Ti 2*p*, Cu 2*p* for NECTO and NSCTO ceramics sintered at different temperature. Meanwhile, atomic ratios of Cu^+^/Cu^2+^ and Ti^3+^/ Ti^4+^ estimated by XPS analysis were shown in the Fig. 4b in order to study the change of variable valence ion content in these ceramic samples. As presented in Fig. 5, two asymmetrically shaped Ti 2*p*_3/2_ and Ti 2*p*_1/2_ are observed, indicating overlapping peaks of Ti^3+^ and Ti^4+^ ions^30,33,38^. According to the Gaussian–Lorentzian profile fitting, four peaks were obtained as displayed in the Fig. 5. This was observed in all sintered ceramics. For the intensive peak Ti 2*p*_3/2_ of NECTO and NSCTO ceramics, binding energy positions defining Ti^3+^ and Ti^4+^ were ~ 456.55–456.98 eV and ~ 457.62–457.96 eV, respectively. Figure 4b shows the radios of Cu^+^/Cu^2+^ or Ti^3+^/Ti^4+^ for NECTO and NSCTO ceramics. It was clearly that the content of Ti^3+^ ions in NECTO and NSCTO ceramics sintered in low temperature (950 °C) maintained a minor amount, and it increased slightly with the increase of sintering temperature.

Simultaneously, the Cu 2*p* spectrum in Fig. 6 indicate the presence of Cu^+^. The main signal corresponding to Cu 2*p*_3/2_ of all ceramic samples could be deconvoluted into two signals: the binding energy positions of Cu^+^ and Cu^2+^ are ~ 932.1–932.56 eV and ~ 933.38–934.03 eV, respectively. That is similar to that reported in the literature for CCTO and TiO_2_-related ceramics^16,39,40^. The appearance of Ti^3+^ and Cu^+^ detected in Na_1/2_Eu_1/2_Cu_3_Ti_4_O_12_ and Na_1/2_Sm_1/2_Cu_3_Ti_4_O_12_ ceramics might have been related to the charge compensation caused by oxygen loss in its lattice structure during the sintering process^5,41^. As shown in the Fig. 4b, the concentration of Cu^+^ in NECTO and NSCTO ceramics samples significantly increased with the rise of sintering temperature, which was different from that of Ti^3+^ in these ceramics.

Simultaneously, it could be clearly confirmed that the content of Cu^+^ in (Na^+^, Eu^3+^) co-doped CCTO ceramics was generally higher than that in (Na^+^, Sm^3+^) co-doped CCTO ceramics for calcium copper titanate related ceramics, which may be related to the different electronic structures of Eu and Sm elements. Obviously, the ionic radius of the low valence state was larger than it of the high valence state. Therefore, the lattice structure of ceramics expands and the lattice parameters increase with the reduction of Cu^2+^ and Ti^4+^ in the grains. The NECTO-1000 and NSCTO-1000 samples with high concentration of Cu^+^ ions had the higher lattice parameters than other ceramics samples, which corresponded to the results observed by XRD.

### Dielectric properties

**Figure 7:**
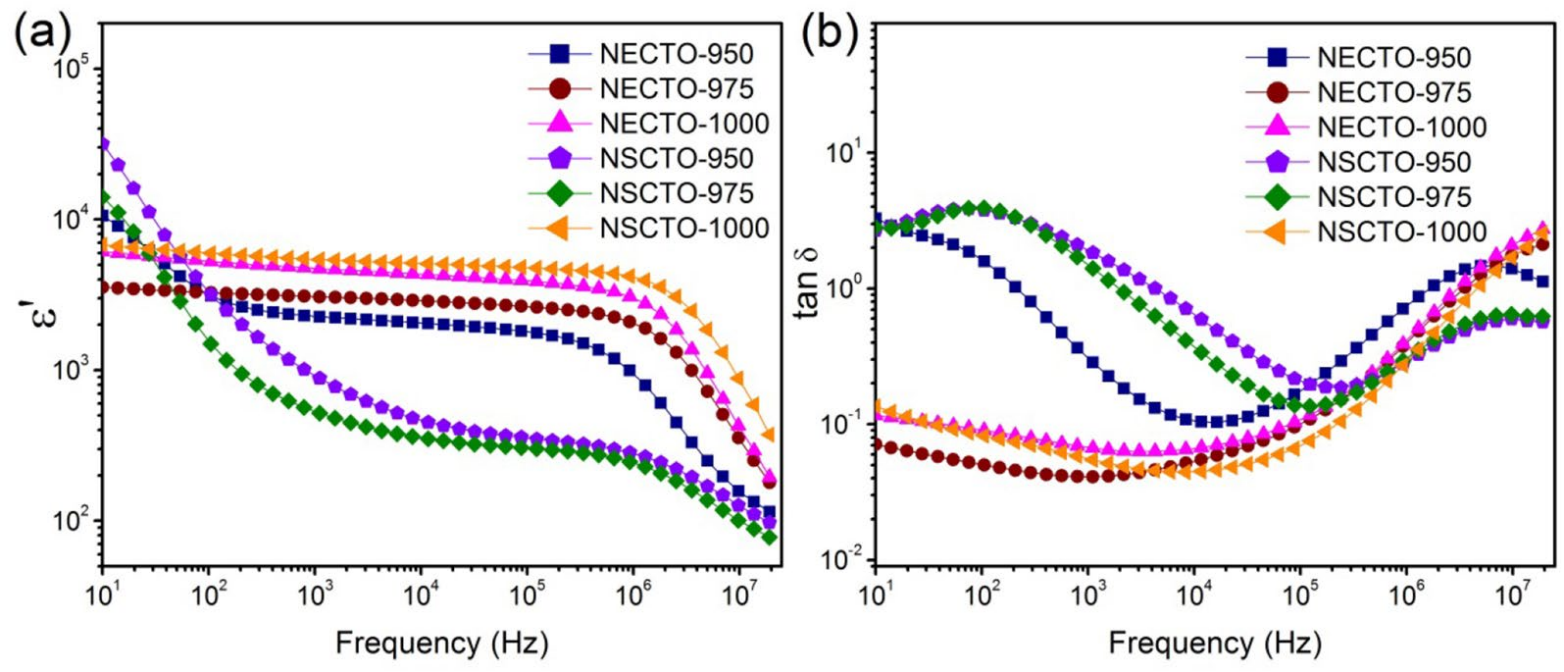
(**a**) Dielectric constant (ε′) and (**b**) dielectric loss (tan δ) at 303 K as a function of frequency for Na_1/2_Eu_1/2_Cu_3_Ti_4_O_12_ and Na_1/2_Sm_1/2_Cu_3_Ti_4_O_12_ ceramics sintered at different temperature.

In order to comprehend how the sintering temperature and mixing ions affects the dielectric responses, frequency dependences of dielectric constant (ε′) and dielectric loss (tan δ) for all samples at room temperature are shown in Fig. 7. Clearly, the overall frequency dependent behaviors of the NECTO and NSCTO ceramics differed obviously. As shown in Fig. 7a, ε′ of NSCCTO-950 sample rapidly decreased from 16,116 to 881 as frequency increased in the low frequency region. This is similar to that observed in NECTO-950 and NSCTO-975 ceramics also prepared at low sintering temperature. However, the permittivity was nearly independent of frequency from 10 to 10^6^ Hz for NECTO-975, NECTO-1000 and NSCTO-1000 samples. Notably, the frequency stabilities of ε′ for NECTO-1000 and NSCTO-1000 samples were better than that of the NECTO-975 sample. The drastic decrease of dielectric constants for these samples at high frequency (> 10^6^ Hz) could be attributed to the interfacial space charge polarization^16^. Figure 7b shows that the tan δ value of the NECTO-975, NECTO-1000 and NSCTO-1000 ceramics in the low frequency were obviously smaller than that of the NECTO-950, NSCTO-950 and NSCTO-975 ceramics. The peaks of dielectric loss (tan δ) for NECTO-950, NSCTO-950 and NSCTO-975 ceramics are presented at low frequencies. The tan δ values at 303 K and 1 kHz of the NECTO-950, NECTO-975, NECTO-1000, NSCTO-950, NSCTO-975 and NSCTO-1100 ceramics were approximately 0.285, 0.041, 0.066, 1.841, 1.407 and 0.055, respectively. Notably, When (Na^+^, Eu^3+^) or (Na^+^, Sm^3+^) was co-doped in CCTO-related ceramic prepared at high sintering temperature (975 or 1000 °C), the ceramic could retain low tan δ value and giant permittivity with good frequency stability.

**Figure 8:**
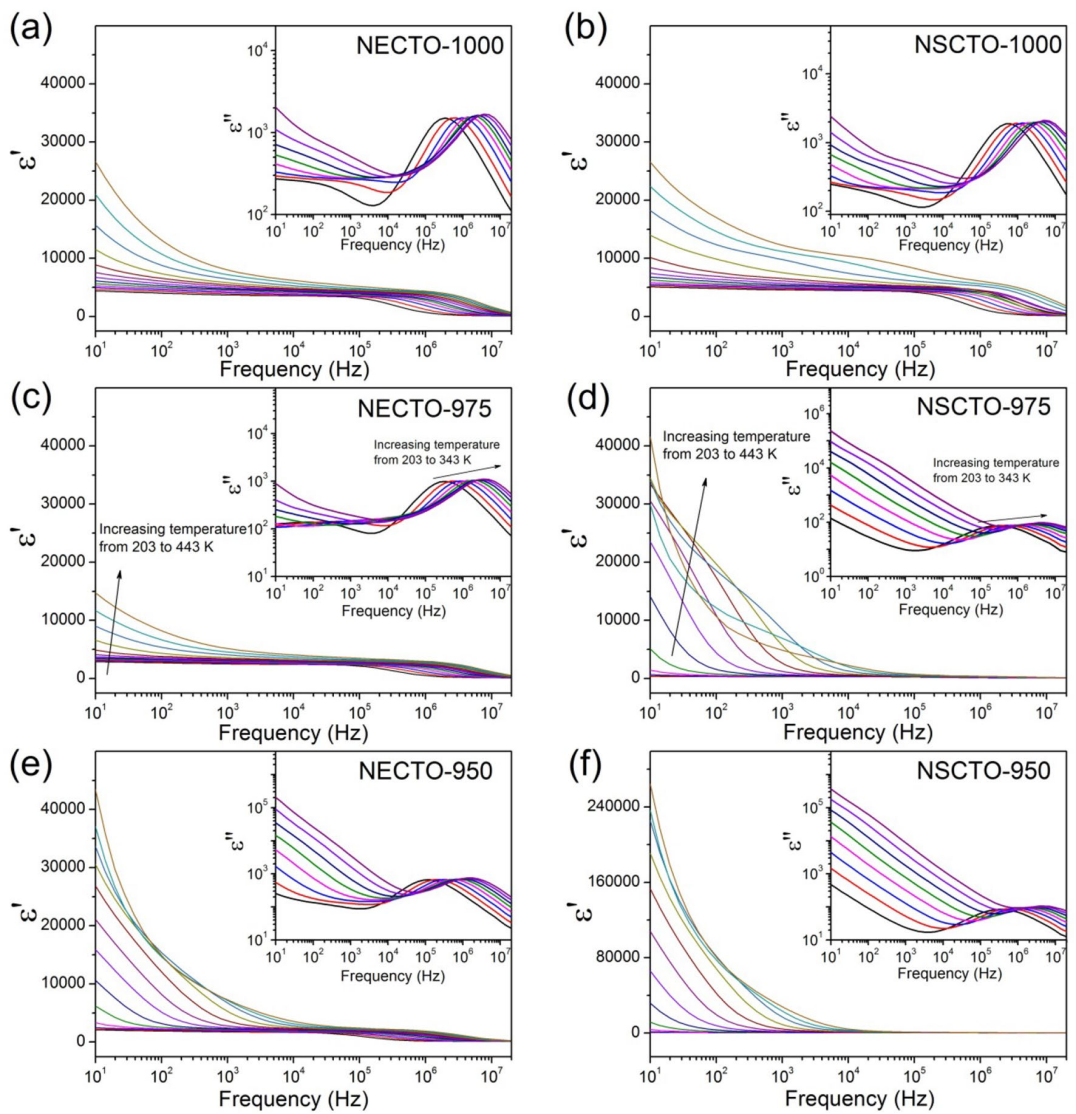
Frequency dependence of ε′ at various temperatures for (**a**) NECTO-1000, (**b**) NSCTO-1000, (**c**) NECTO-975, (**d**) NSCTO-975, (**e**) NECTO-950 and (**f**) NSCTO-950; their insets show the frequency dependence of ε″ in a temperature range from 203 to 343 K.

The frequency of real part ε′ and imaginary part ε″ for NECTO and NSCTO ceramics at different temperature were shown in the Fig. 8 and its insets. Three plateau characteristics in the plots of ε′ versus frequency were observed in the temperature range from 203 to 443 K, which corresponded to three main parts of dielectric responses, respectively. The first plateau in a low frequency range was observed at the high temperature, which is attributed to an sample-electrode interface response^21,36^. In the low frequency region, as the test temperature increased, the dielectric constant changed more sharply with frequency, indicating that the sample-electrode interface response, i.e. the surface barrier layer capacitor (SBLC) polarization mechanism, was more obvious. Therefore, as shown in Fig. 8, there was a strong SBLC effect inside the NECTO-950, NECTO-975 and NSCTO-950 ceramics prepared at low sintering temperature, which leads to the abnormal high dielectric constant of these ceramics in the low-frequency region (shown in the Fig. 7a). As the Fig. 7b, the dielectric loss peaks of these three ceramics observed at low frequencies were also related to the SBLC effect. The primary plateau observed at intermediate frequency was attributed to the dielectric response of the grain boundaries^27,42^, which was evident in the NECTO-1000, NSCTO-1000, and NECTO-975 ceramics prepared at high sintering temperature. However, the high frequency plateau observed at the low testing temperature was originated from the bulk response^23^. The dielectric responses of these three parts were clearly observed in CCTO-related ceramics^27,42,43^. As shown in the Fig. 8 insets, the peak of ε″ shifted to higher frequencies with increasing temperature, indicating that the dielectric relaxation process was thermally activated. It was found that the high frequency relaxation activation energies of the NECTO-950, NECTO-975, NECTO-1000, NSCTO-950, NSCTO-975, NSCTO-1000 ceramics were 0.116, 0.106, 0.102, 0.114, 0.113 and 0.100 eV, respectively. These activation energies were related to the electric response of the bulks or grains, which were similar to other CCTO-like ceramics^44–46^.

### Impedance spectroscopy analysis

**Figure 9:**
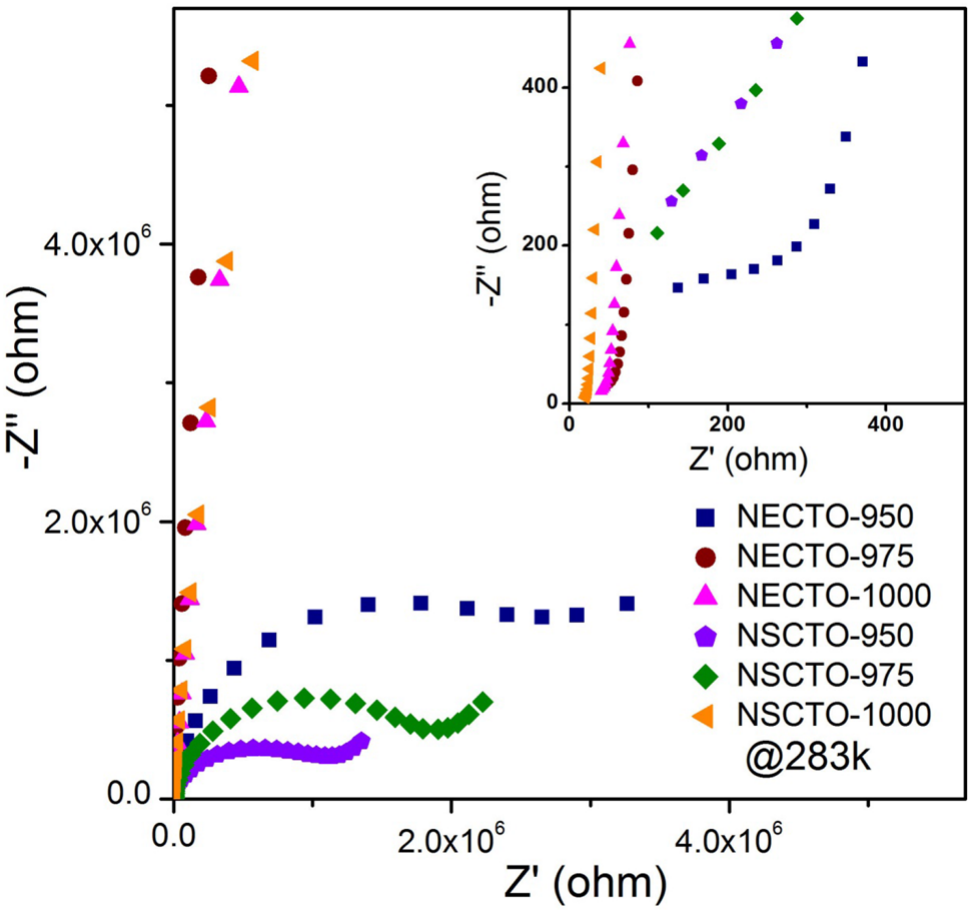
Impedance complex plane (Z*) plot at 283 K for Na_1/2_Eu_1/2_Cu_3_Ti_4_O_12_ and Na_1/2_Sm_1/2_Cu_3_Ti_4_O_12_ ceramics with different sintering temperature. Inset shows an expanded view of the Z* plot at 283 K in high frequency range.

To describe the dielectric behaviors of these ceramics, the impedance spectroscopy for NECTO and NSCTO ceramics was employed to illuminate this. In Fig. 9 and its inset, the linear part of the semicircular arcs with nonzero intercept on the Z′ axis was observed at 283 K. However, the semicircular arcs became apparent in low frequency region for NECTO-950, NSCTO-950 and NSCTO-975 ceramics (Fig. 9). For, CCTO-related ceramics, these electrical response are associated with the contributions from grain boundaries and grains, respectively^47^.

The resistance values of grain, grain boundary and surface for NECTO and NSCTO ceramics were estimated using the equivalent R(RQ) circuit^23^ shown in Table 2. As displayed in the Table 2, the grain resistance (R_g_) values of these Na_1/2_Ln_1/2_Cu_3_Ti_4_O_12_ (Ln = Eu or Sm) ceramics evidently decreased with increasing sintering temperature. The variation of R_g_ values for these ceramics was related to the appearance of low valent ions (Cu^+^ and Ti^3+^) at the grain during high-temperature sintering process^14,21,31^.

**Table 2:** Grain resistance (R_g_), grain boundary resistance (R_gb_), surface resistance (R_s_) and various activation energies (*E*_g_ for grain, *E*_gb_ for grain boundary and *E*_s_ for surface) data for Na_1/2_Eu_1/2_Cu_3_Ti_4_O_12_ and Na_1/2_Sm_1/2_Cu_3_Ti_4_O_12_ ceramics prepared at different temperature.

Sample	Sintering temperature (°C)	*R*_g_ (Ω)	*R*_gb_ (Ω)	*R*_s_ (Ω)	*E*_g_ (eV)	*E*_gb_ (eV)	*E*_s_ (eV)
NECTO	950	338	3.11 × 10^6^	2.76 × 10^6^	0.110	0.480	0.454
	975	68.2	2.44 × 10^8^	–	0.0920	0.747	–
	1000	48.2	2.34 × 10^8^	–	0.0868	0.739	–
NSCTO	950	1091	8.12 × 10^5^	7.9 × 10^5^	0.0947	0.502	0.648
	975	961	1.79 × 10^6^	1.39 × 10^6^	0.0942	0.493	0.579
	1000	34.3	9.51 × 10^7^	–	0.0837	0.783	–

The resistance data were obtained by fitting the impedance spectra in Fig. 9 using equivalent R(RQ) circuit.

Combined with XPS data, the Cu^+^ and Ti^3+^ ions were prone to occur in ceramics due to the occupation of multiple cations (Na^+^, Eu^3+^) or (Na^+^, Sm^3+^) in CCTO-related ceramics. The higher the sintering temperature, the higher the concentration of low-valence ions (Cu^+^ and Ti^3+^) in NECTO or NSCTO ceramic (shown in the Fig. 4b), and the higher the conductivity of the grain. Hence R_g_ of NECTO-975, NECTO-1000 and NSCTO-1000 samples was much smaller than that of the other samples. The semiconducting conduction of grain in these samples was highly related with the electron hopping between Cu^+^ ↔ Cu^2+^ and Ti^3+^ ↔ Ti^4+^. However, we have found that R_gb_ values of the Na_1/2_Ln_1/2_Cu_3_Ti_4_O_12_ (Ln = Eu or Sm) ceramics increased significantly with the rise of sintering temperature. The insulation of grain boundaries may be related to the enrichment of Cu at grain boundaries in these ceramics, which has been observed in other ACTO-type ceramics^8,15,35,48^. Moreover, the high insulating grain boundaries could reduce the dielectric loss of the ceramic materials^14^. So the NECTO-975 ceramic with the highest grain boundary resistance in the NECTO system had the lowest dielectric loss about 0.041 at 303 K and 1 kHz (observed in Fig. 7b). Similarly, the NSCTO-1000 ceramic in the NSCTO series also had the lowest dielectric loss about 0.055 (at 303 K and 1 kHz). It was clearly demonstrated that R_g_ was more than 5 orders of magnitude smaller than R_g_ for NECTO-975, NECTO-1000 and NSCTO-1000 samples. Hence this suggests that the origin of giant dielectric response of these ceramics sintered in high temperature was correlated with a typical IBLC polarization mechanism.

**Figure 10:**
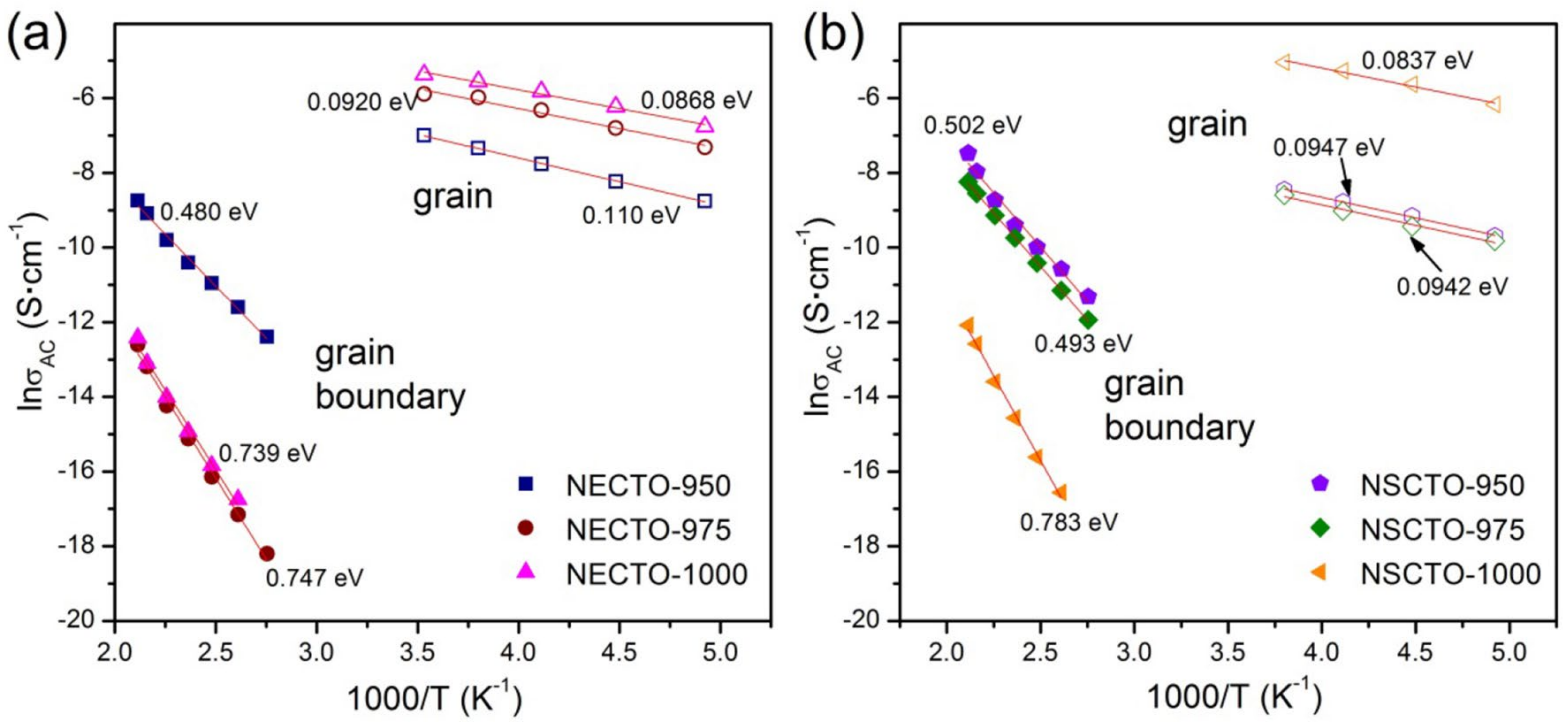
Arrhenius plots for the temperature dependence of σ_AC_ for (**a**) NECTO and (**b**) NSCTO samples. Red solid lines are the linear fitting curves.

To optimize the results, the temperature dependence of AC conductivities (σ_AC_) was measured in the temperature range of 103–473 K for NECTO and NSCTO ceramics, as illustrated in Fig. 10. Moreover, the activation energy required for the AC conductivities of charge carriers in the grains and grain boundaries can be calculated using the Arrhenius law^41^:

1 $$\sigma_{AC} = \sigma_{o} {\text{exp}}\left( { - E_{a} /k_{B} T} \right)$$

where *σ*_AC_ is the AC conductivity, *σ*_o_ is the pre-exponential term, *E*_a_ is the activation energy, and *κ*_B_ (J/K) and *T* are the Boltzmann constant and absolute temperature (K), respectively. According to Eq. (1), the values of activation energies (*E*_g_ for grain, *E*_gb_ for grain boundary and *E*_s_ for surface) can be calculated by fitting the curves in Fig. 10. Both E_g_ and *E*_gb_ values of NECTO ceramics had different trends with the change of sintering temperature, as displayed in the Fig. 10a. The E_g_ of NECTO-950, NECTO-975 and NECTO-1000 ceramics were 0.110, 0.092 and 0.0868 eV, respectively. A slight decrease in E_g_ is consistent with the increase of grain conductivity shown in the Table 2. Meanwhile, with the increase of sintering temperature, the E_gb_ values of NECTO ceramics were 0.480, 0.747 and 0.739 eV, respectively. As shown in the Fig. 10b, the observed E_g_ values of NSCTO-900, NSCTO-975 and NSCTO-1000 ceramics were 0.0947, 0.0942 and 0.0837 eV, respectively, while the observed E_gb_ values were 0.502, 0.493 and 0.783 eV, respectively. The E_g_ values of NECTO and NSCTO ceramics were basically similar to the values of high-frequency relaxation activation energy obtained in Fig. 8, which further indicated that the dielectric response in the high frequency region was related to the grain. The changing trend of E_g_ and E_gb_ values of NSCTO samples with sintering temperature was very similar to that of NECTO samples. Compared with NECTO-950, NSCTO-950 and NSCTO-975 ceramics sintered at low temperature, E_gb_ and E_g_ values of the other ceramics prepared at high sintering temperature differed significantly, indicating that the IBLC polarization mechanism was formed, which will caused the giant dielectric response observed in NECTO-975, NECTO-1000 and NSCTO-1000 ceramics. The calculated E_gb_ and E_g_ values were nearly same as other reported in CCTO and TiO_2_-related ceramics^29,49–51^.

**Figure 11:**
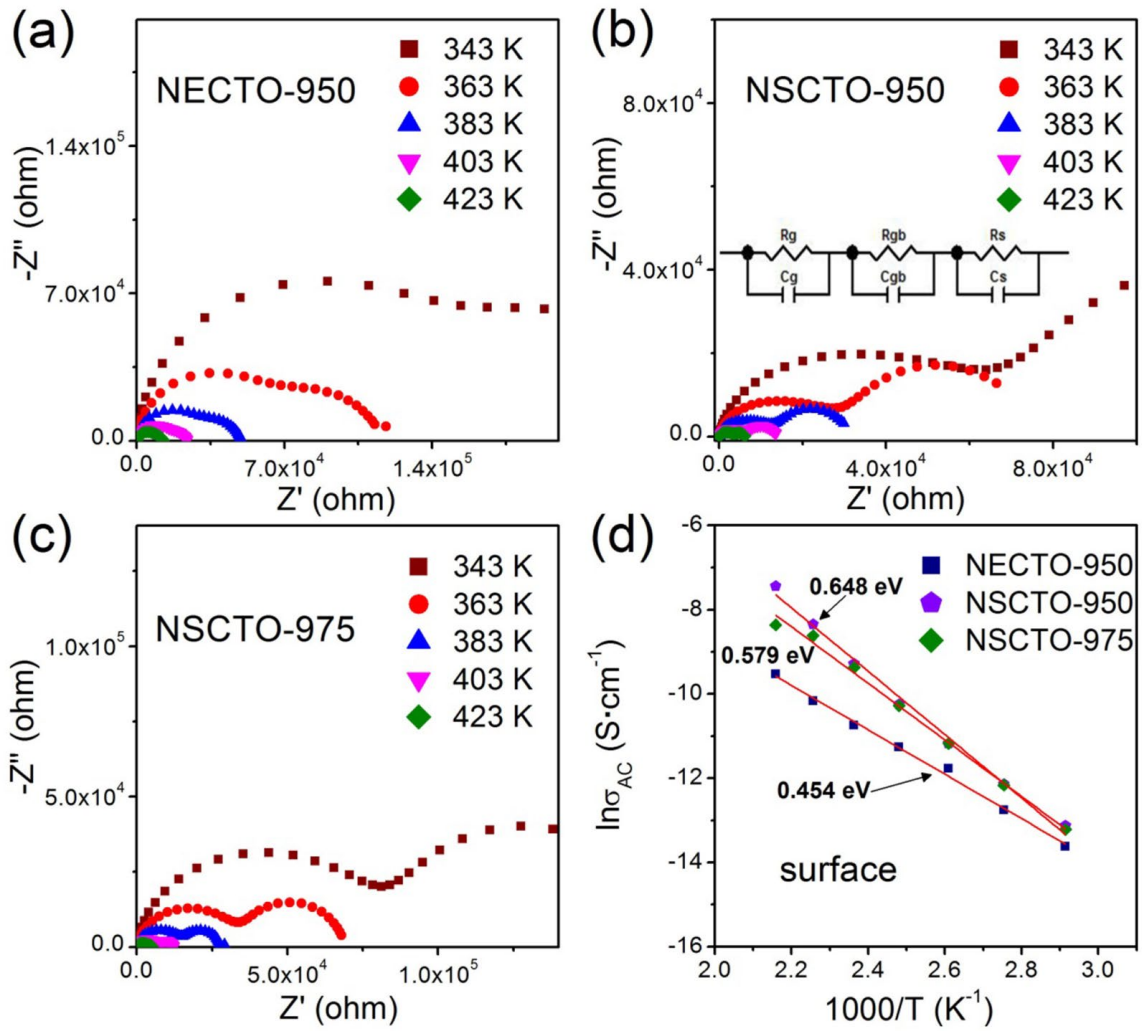
Impedance complex plane plots in the temperature range of 343–423 K for (**a**) NECTO-950, (**b**) NSCTO-950 and (**c**) NSCTO-975 ceramics; (**d**) Arrhenius plots of ac conductivity (σ_AC_) for these three samples. Red solid lines are the linear fitting curves. Equivalent circuit indicated in frame (**b**) is used to represent the electrical properties of grain, grain boundary and surface effects.

However, the abnormal high dielectric constant and dielectric loss observed in the low frequency for NECTO-950, NSCTO-950 and NSCTO-975 ceramics (shown in the Fig. 7) cannot be attributed to the IBLC effect. According to the analysis of Fig. 8, it could be governed by the polarization mechanism of SBLC. To confirm the possible source of the abnormal dielectric response in these ceramics, impedance complex spectrum of variable temperature (343–423 K) was displayed in the Fig. 11a–c, respectively. Because polycrystalline ceramic materials generally exhibit grain, grain boundary or surface impedances^23^, they can be represented by the equivalent circuit shown in Fig. 11b. Meanwhile, the Arrhenius plots of ac conductivity (σ_AC_) for these three samples displayed in the Fig. 11d. It was observed that a new diameter of the semicircle arcs of the NECTO-950, NSCTO-950 and NSCTO-975 samples in the low frequency decreased with increasing temperature, which was a thermal activation process. As displayed in the Fig. 11d and Table 2, the activation energy of Na_1/2_Eu_1/2_Cu_3_Ti_4_O_12_ and Na_1/2_Sm_1/2_Cu_3_Ti_4_O_12_ ceramics sintered at 950 or 975 °C were 0.454, 0.648 and 0.579 eV, respectively. Considering the activation energy obtained in Fig. 11d, this thermal excitation process at low frequency region did not represent the electrical response of the grain or grain boundary, but was caused by the surface effect^12,36,38^. It was indicated that abnormal dielectric response for NECTO-950, NSCTO-950 and NSCTO-975 ceramics could be attributed to interfacial polarization based on SBLC effect. This was also consistent with the result obtained in Fig. 8. Interestingly, the doping of (Na^+^, Eu^3+^) or (Na^+^, Sm^3+^) in CCTO lattice could induce the appearance of Cu^+^ and Ti^3+^ in the grain during the sintering process. The low concentration of variable valence ions in the lattice could result in a small conductivity inside the grain at relatively low sintering temperature (950 °C). The abnormal permittivity and dielectric loss was contributed from the SBLC polarization mechanism for NECTO-950 and NSCTO-950 ceramics. NECTO and NSCTO ceramics prepared at high sintering temperature (1000 °C) would have more charge carriers with the increase of Cu^+^ and Ti^4+^ ions in ceramic structure, resulting in the formation of semiconductive region inside the grain. So, the giant dielectric constant and low dielectric loss for NECTO-1000 and NSCTO-1000 ceramics had been derived from an IBLC effect. According to the conclusion of XPS, the doping of (Na^+^, Eu^3+^) in CCTO-based ceramic was more likely to produce Cu^+^ ions than the doping of (Na^+^, Sm^3+^) in these ceramic. Therefore, the IBLC polarization mechanism appeared in the NECTO-975 ceramic containing high concentration of Cu^+^, and the ceramic showed good dielectric stability. On the contrary, SBLC polarization mechanism appeared in NSCTO-975 with abnormal dielectric response, while Cu^+^ ion was relatively low in the ceramic. In summary, the number of charge carriers (vacancies or free electrons) in the ceramic lattice will impress the conductivity of the grains, resulting in a competitive coexistence between SBLC and IBLC effects, thereby affecting the dielectric properties of ceramic.

## Conclusion

This work systematically dealt with the structural, ion valence and dielectric properties of Na_1/2_Eu_1/2_Cu_3_Ti_4_O_12_ and Na_1/2_Sm_1/2_Cu_3_Ti_4_O_12_ ceramics sintered at different temperature. According to the XRD and RS data, all ceramics showed a single phase of CCTO-related ceramics. The substitutions of (Na^+^, Eu^3+^) or (Na^+^, Sm^3+^) in the CCTO lattice could induce the appearance of Cu^+^ and Ti^3+^ in grain. The higher sintering temperature, the more content of Cu^+^ and Ti^3+^, and the higher conductivity of grain in Na_1/2_Eu_1/2_Cu_3_Ti_4_O_12_ and Na_1/2_Sm_1/2_Cu_3_Ti_4_O_12_ ceramics, which also led to the gradual transformation of the polarization mechanism inside the ceramics from SBLC to IBLC. Therefore, Na_1/2_Eu_1/2_Cu_3_Ti_4_O_12_ and Na_1/2_Sm_1/2_Cu_3_Ti_4_O_12_ ceramics sintered at 1000 °C had giant dielectric permittivity of about 10^4^, a low dielectric loss of about 10^−2^ and good frequency stability of about 10^2^–10^6^ Hz. At the same sintering temperature, the doping of Eu ions in the Na_1/2_Ln_1/2_Cu_3_Ti_4_O_12_ system would induce from more Cu^+^ ions inside the ceramics than the doping of Sm ions during the process of preparation. This resulted in a more pronounced IBLC polarization mechanism in Na_1/2_Eu_1/2_Cu_3_Ti_4_O_12_ ceramics than in Na_1/2_Sm_1/2_Cu_3_Ti_4_O_12_ ceramics. Therefore, Na_1/2_Eu_1/2_Cu_3_Ti_4_O_12_ ceramic prepared at 975 °C had better dielectric stability than Na_1/2_Sm_1/2_Cu_3_Ti_4_O_12_ ceramic sintered at same temperature.

## Data Availability

All data generated or analysed during this study are included in this published article.
